# Mixing of Excitons in Nanostructures Based on a Perylene Dye with CdTe Quantum Dots

**DOI:** 10.3390/ma16020552

**Published:** 2023-01-06

**Authors:** Yuri P. Piryatinski, Markiian B. Malynovskyi, Maryna M. Sevryukova, Anatoli B. Verbitsky, Olga A. Kapush, Aleksey G. Rozhin, Petro M. Lutsyk

**Affiliations:** 1 Institute of Physics, National Academy of Sciences of Ukraine, 46 Prospekt Nauky, 03680 Kyiv, Ukraine; 2 V. Lashkaryov Institute of Semiconductors Physics, National Academy of Sciences of Ukraine, 41 Prospekt Nauky, 03680 Kyiv, Ukraine; 3 Aston Institute of Photonic Technologies, College of Engineering and Physical Sciences, Aston University, Aston Triangle, Birmingham B4 7ET, UK

**Keywords:** perylene dye, nanoparticles, quantum dots, cadmium telluride, photoluminescence, time-resolved spectroscopy, exciton

## Abstract

Semiconductor quantum dots of the A_2_B_6_ group and organic semiconductors have been widely studied and applied in optoelectronics. This study aims to combine CdTe quantum dots and perylene-based dye molecules into advanced nanostructure system targeting to improve their functional properties. In such systems, new electronic states, a mixture of Wannier–Mott excitons with charge-transfer excitons, have appeared at the interface of CdTe quantum dots and the perylene dye. The nature of such new states has been analyzed by absorption and photoluminescence spectroscopy with picosecond time resolution. Furthermore, aggregation of perylene dye on the CdTe has been elucidated, and contribution of Förster resonant energy transfer has been observed between aggregated forms of the dye and CdTe quantum dots in the hybrid CdTe-perylene nanostructures. The studied nanostructures have strongly quenched emission of quantum dots enabling potential application of such systems in dissociative sensing.

## 1. Introduction

Self-assembly of molecules and the formation of one-dimensional molecular structures with an atomically close distance between the molecules in one direction attracts considerable attention in recent decades [1,2,3]. For example, planar aromatic molecules organize into one-dimensional face-to-face stacks with a strong intermolecular overlap of π-orbitals. Such structures are interesting for applications in photoelectronic devices such as solar cells [4], light-emitting diodes and transistors [5], etc. Perylene derivatives, such as N,N-dimethylperylene-3,4,9,10-dicarboximide (MePTCDI), or 3,4,9,10-perylenetetracarboxylic dianhydride (PTCDA), are well-known examples of materials forming one-dimensional crystals with an extremely small distance between molecular planes in one-dimensional stacks (3.37 Å for PTCDA, 3.40 Å for MePTCDI) [6,7]. The properties of perylene derivatives crystals are similar to features of both conventional inorganic semiconductors and organic molecular crystals. Therefore, perylene-based molecular structures are an ideal model material to study fundamental excitonic processes linking inorganic and organic semiconductor classes of materials that may ultimately prove useful for applications in optoelectronic devices.

Inorganic semiconductor nanoparticles or quantum dots (QDs) of the A_2_B_6_ group, (CdS, CdTe) have wide practical applications in optoelectronics, for example as labels for biological research [8,9,10]. Combining two objects, such as CdTe QDs and perylene dyes, into one system has the potential to significantly improve their functional properties, therefore a comprehensive understanding of their fundamental properties is needed. In such systems, new (mixed) electronic and excitonic states may appear at the interface of organic and inorganic materials. Various models of excitons are used to classify excitons: small-radius Frenkel exciton (FE) model [11,12], charge transfer exciton (CTE) model [13,14,15], and the large-radius Wanier-Mott exciton (WME) model [16]. FE and CTE models are used to classify excitons in organic semiconductor materials, while the WME model is typical for inorganic semiconductors. FE is a neutral excited state in which an electron and a hole are located on the same molecule. The intermolecular interaction leads to a finite transition integral for the transfer of electronic excitation from one molecule to another, and as a result, FEs propagate through a crystal as coherent waves. CTE consists of a pair of charge carriers located on different neighboring molecules. Such arrangements are ensured in organic crystals because (in contrast to inorganic semiconductors) the binding energy of lower CTEs is greater than the width of the valence and the conduction bands. WME model considers the Coulomb interaction between an electron and a hole and is based on an approximation of the effective mass for them in a periodic lattice potential. The main characteristic of WME is hydrogen absorption and emission behavior in crystals with a large dielectric constant. The average distance between the electron and the hole for this type of exciton is much larger than the lattice constant. Mixing the different excitons is an appealing area of fundamental research allowing one to pursue enhancement of resonant optical nonlinearity, fluorescence efficiency and relaxation processes [17].

**Figure 1:**
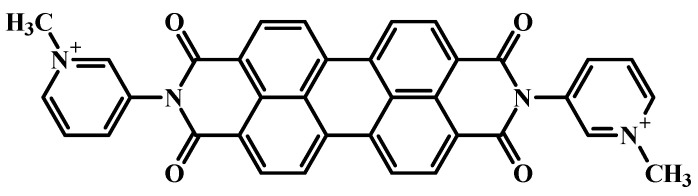
The structural formula of the perylene-3,4:9,10-bis(dicarboximide)-N,N-bis(1-methyl-3pyridinium) bis-n-toluenesulfonate (2416SL) molecule.

The studied perylene derivative, perylene-3,4:9,10-bis(dicarboximide)-N,N-bis(1-methyl-3pyridinium) bis-n-toluenesulfonate (2416SL) is similar in its structural and electronic properties to the well-investigated PTCDA. The difference is that 2416SL molecules have ionic groups on the periphery (Figure 1), which makes them soluble in water [18], therefore semiconductor crystalline structures with 2416SL can be prepared from solutions. In water, 2416SL molecules associate into aggregates, which at high concentrations assemble into structures with long-range orientational order. Such structures can form lyotropic chromonic liquid crystals (LCLC), the elementary building blocks of the metaphase of which are elongated disk-like molecular aggregates [19]. 2416SL can form oriented nanostructured films preserving the LCLC orientational order in solid crystalline film [18,20,21]. The main motivation for researching 2416SL in different aggregated states stems from promising excitonic and related optical properties, which appear due to the regular planar organization of 2416SL molecules in thread-like H-aggregates having π-π stacking with an unusually small intermolecular distance of 0.34 nm [6,7]. The excitonic properties of neat 2416SL have not been studied comprehensively, and this aspect will be investigated here as well in more detail. Thus, 2416SL is a relevant model object that allows us to study a wide range of fundamental phenomena involved in the operation of various electronic devices made of organic and inorganic materials. Functionalizing CdTe QDs by perylene dye, such as 2416SL, will allow one to create nanostructures with novel features, and fundamental spectral characteristics of such new nanostructures have to be analyzed. The study of the nature of exciton states in the bulk and at the interface of organic/inorganic materials is one of the fundamental objectives for the application of such hybrid functional systems in the future.

## 2. Materials and Methods

The structural formula of the studied 2416SL molecule is shown in Figure 1. 2416SL was synthesized at the Institute of Organic Chemistry, the National Academy of Sciences of Ukraine using the methodology described before [20]. Solutions of 2416SL were prepared in dimethyl sulfoxide (DMSO) and water. 2416SL dissolves well in DMSO. In water, even at low concentrations, 2416SL molecules aggregate. The aggregation of 2416SL molecules in aqueous solvents can be associated with the high hydrophobicity of their perylene core-chromophore [22]. The aqueous solutions in water were heated to 90 °C and then cooled down before measurement. The concentrations of the solutions were in the range of (10^−3^–5·10^−6^) M.

2416SL films were obtained by drop casting of high concentration (0.1 M) aqueous solution on a quartz substrate and drying at room temperature. The films were annealed at 470 K to improve crystalline structure.

A dispersion of CdTe quantum dots (QDs) in deionized water was obtained in the presence of thioglycolic acid (TGA) [10]. All reagents and solvents obtained from commercial suppliers were of reagent grade quality. Milli-Q water, CdI_2_, NaOH, and thioglycolic acid (TGA ≥ 90%) were purchased from Himlaborreactive (Ukraine). In the synthesis of the QDs, each chemical element was introduced into the reactor in the form of a precursor: a molecule or complex containing at least one constituent element. In our case, the Cd_2_^+^ source was the CdI_2_ salt, and the Te_2_^−^ source was H_2_Te gas prepared electrochemically in a galvanostatic cell. The low-temperature colloidal synthesis has been performed in the reactor of complete mixing in the presence of TGA as a stabilizer. CdI_2_ was dissolved in water, and TGA was added under stirring, followed by adjusting the pH to 10 by dropwise addition of NaOH solution. H_2_Te gas was passed through the solution using argon as a carrier gas. The size of CdTe QD increased with the duration of the synthesis. The size of CdTe QDs was determined by dynamic light scattering and the ratio of the particle diameter, *d*, and the absorption wavelength of the first exciton maximum. For studied CdTe QDs, the average diameter *d* was 2.5 and 3.5 nm, having a relatively narrow distribution of QDs sizes characterized by dynamic light scattering (for *d* = 2.5 nm, the distribution range is 1.5–6.0 nm, and 3.5 nm QDs sizes spread slightly wider over 1.8–9.0 nm). The concentration of CdTe QDs in the initial dispersions was approx. 10^−5^ M.

The formation of hybrid nanostructures of CdTe-2416SL took place by admixing initial dispersions of CdTe in portions of *V_n_* = 0.1·*n* mL, where *n* was taken from 1 to 15, to 1 mL of an aqueous solution of 2416SL (with a concentration of 5·10^−5^ M). The mixture of CdTe-2416SL at *n* = 15 has been studied in two forms due to abundant aggregation and precipitate formation. One form was a CdTe-2416SL supernatant where all precipitate was allowed to go down for 24 h and only the top half of the mixture was studied. Another form was a freshly mixed CdTe-2416SL with all the micro and nano-aggregations present in the dispersion.

The structure of electron-vibrational and excitonic transitions in the studied samples (2416SL solutions, CdTe QDs dispersions, 2416SL films, and hybrid systems based on CdTe-2416SL) was studied by analyzing electronic absorption spectra, steady-state, and time-resolved photoluminescence (PL) spectra. The complex use of spectral techniques allowed us to identify molecular and exciton signatures in the studied systems.

Absorption spectra were measured using a Lambda 1050UV/VIS/NIR spectrophotometer (PerkinElmer, US). Steady-state PL spectra were measured using a USB2000+UV-VIS-ES spectrometer through an optical fiber with a diameter of 600 μm. LLS-385 LED (Ocean Optics, US) and EPL-405 laser (Edinburgh Instruments Ltd., Livingston, UK) were used to excite steady-state PL with the corresponding *λ*_e_ wavelength.

Time-resolved PL emission spectra (TRES) were measured using a LifeSpec II spectrofluorimeter (Edinburgh Instruments Ltd., Livingston, UK). An EPL-405 laser with a wavelength of *λ*_e_ = 405 nm and a pulse duration of 40 ps was used to excite time-resolved PL in the visible range. The frequency of excitation pulses can be adjusted in the range of 10 kHz–20 MHz. To determine the lifetimes *τ* of excited states of molecules, the time-correlated photon counting with picosecond time resolution was used allowing us to measure PL decay kinetics of weakly emitting samples with characteristic lifetimes of (10^−6^–10^−11^) s. To excite PL in this method, a sequence of short excitation pulses of radiation from the lasers with a strictly fixed follow-up period is used. The probability of PL detection is kept below one photon when the object is excited by a single pulse, and the repetition frequency of the exciting pulse is set as high as possible. On the other hand, the sequence of pulses is maintained in such a way that the time interval between pulses is at least 5–10 times longer than the decay time of PL being recorded. The obtained time dependence of PL kinetics, *I*(*t*), was approximated by the expression:

$$I\left(t\right)=IRF*\overset{n}{\underset{i=1}{\sum }}A_{i}\exp(-\frac{t}{\tau_{i}})$$

where *IRF* is the instrument response function of the detector, *i* is a serial number, *τ* is the lifetime of the excited state, and *A_i_* is the weighting factor. To measure *IRF*, the certified colloidal LUDOX solution was used.

To establish the true PL attenuation curve according to experimental data, it is necessary to solve the integral equation $I\left(t\right)=\int_{0}^{t}F\left(t-t^{\prime }\right)G\left(t^{\prime }\right)dt^{\prime }$, where *I*(*t*) is the experimental dependence of PL intensity on time, *F*(*t*) is the true dependence of PL attenuation on time, *G*(*t*) is the *IRF*. Using a sequence of PL kinetic curves for different emission wavelengths, TRES maps were constructed representing the spectral dependence of PL on the delay time, *t*. TRES map is a functional dependence of the PL intensity on two variables—the radiation wavelength (*λ_EM_*; Y-axis) and the delay time (*t*; X-axis). In the TRES map, as instantaneous PL spectra are measured, the corresponding *t* are recorded with the reference to the maximum of the laser pulse in the *IRF*, when *t* = 0 ns. To calculate *τ* and plot the TRES maps, the F900 software package (version 7.2, Edinburgh Instruments Ltd., Livingston, UK) was used.

## 3. Results and Discussion

### 3.1. Solutions and Films of 2416SL

**Figure 2:**
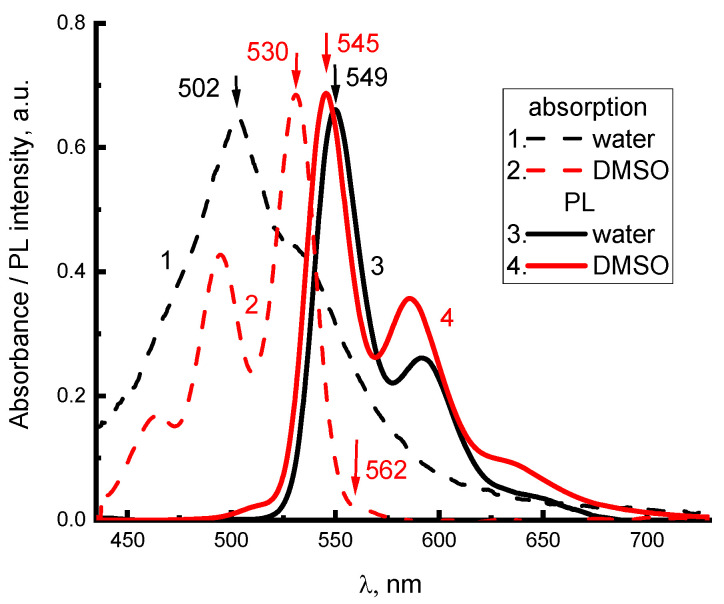
Spectra of absorption (1,2) and steady-state PL (3,4) of molecular solutions of 2416SL in DMSO (2,4) and water (1,3) at the concentration of 5·10^−6^ M, *λ*_e_ = 385 nm, T = 296 K.

To determine the structure of electron-vibrational and exciton transitions in 2416SL, the absorption and PL spectra of aqueous and DMSO solutions at different concentrations were studied. Furthermore, the spectra of 2416SL films deposited on quartz provided additional insight. Absorption (Figure 2, curves 1,3) and PL (Figure 2, curves 2,4) spectra for low concentration (5·10^−6^ M) solutions of 2416SL in water and DMSO at 296 K have variations, which can be associated with their different solubility. The positions of the maxima of the electronic and electronic-vibrational bands in the absorption and PL spectra (Figure 2) of solutions of 2416S in water and DMSO are summarized in Table 1 and Table 2.

**Figure 3:**
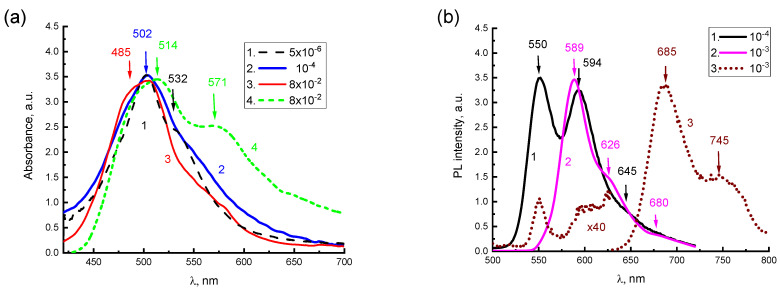
(**a**) Absorption spectra of aqueous solutions for 2416SL at the concentrations of 5·10^−6^ M (1), 10^−4^ M (2), and 8·10^−2^ M (3,4); *λ*_e_ = 405 nm; T = 296 K (1–3) and 77 K (4). (**b**) Steady-state PL spectra of aqueous solutions for 2416SL at the concentrations of 10^−4^ M (1) and 10^−3^ M (2,3); *λ*_e_ = 405 nm; T = 296 K (1,2) and 4.2 K (3).

The spectra in DMSO (Figure 2) are mirror symmetric, have a Stokes shift of 530 cm^−1^, and oscillating repetitions with a frequency close to 1330 cm^−1^ forming solid evidence that the spectra are of molecular origin. Absorption spectra for aqueous solutions of 2416SL (Figure 2 and Figure 3a) are lacking mirror symmetry between the absorption and PL spectra. There is a significantly reduced light absorption in the region of the purely electronic S_0_(0) → S_1_(0) optical transition, and a high intensity 502 nm band, approximately in the region of S_0_(0) → S_1_(1) transition. In the present work, only aqueous solutions of 2416SL are studied in detail, because aqueous solutions of CdTe and their mixtures with 2416SL are the focus of this study. The absence of mirror symmetry between the absorption and PL spectra of aqueous solutions, additional absorption in the short wavelength region, and reduced contribution of molecular spectral signatures evidence that, even at low concentrations, H-aggregates are formed. Therefore, the absorption and PL spectra are formed not only by molecular but also by collective excitations in the aggregates, and disk-like molecules of 2416SL aggregate into thread-like columnar structures with a diameter equal to the size of the molecule. Furthermore, the perylene derivatives are well-known for forming H-aggregates, where molecules are positioned almost parallel to each other [23]. Such one-dimensional molecular aggregates have features characteristic of collective excitations (excimers, FE [11,12], CTE [24,25,26]).

Due to the stacking parallel orientation of molecules and their dipoles in molecular H-aggregates in solutions and films, their PL of FE is significantly quenched in comparison to an isolated molecule. This is happening because optical transitions for FE in H-aggregate absorption and PL between S_0_(0) ↔ S_1_(0) electronic states are forbidden [16]. However, the strict prohibition of S_0_(0) ↔ S_1_(0) transitions is valid, only for excitation delocalized along an infinite crystal [16]. In real crystals, the effect of dipole ordering depends on the exciton coherence length. In molecular aggregates and thin polycrystalline films, the coherence length decreases due to thermal and structural disorder [26,27,28], and optical S_1_(0) → S_0_(1) transitions to higher electronic vibrational states of the S_0_(1) ground state are allowed, albeit with a smaller intensity. This can be related to the characteristic features of the absorption spectra for aqueous solutions of 2416SL at different concentrations (Figure 3), there is a lack of mirror symmetry between the electronic absorption and PL spectra at room temperature, as well as very low absorption in the region of the purely electronic S_0_(0) → S_1_(0) transition and a significant intensity of the 502 nm band, approximately in the region of the allowed S_0_(0) → S_1_(1) transition. The above features of absorption and PL spectra for aqueous solutions of 2416SL can be explained by the manifestation of FE in H-aggregates, which are formed in these solutions.

For aqueous solutions of 2416SL of different concentrations, starting from 5·10^−6^ M and more, changes in the PL spectra at room temperature are much more significant than in the absorption spectra (Figure 3). Two bands with maxima at 550 and 594 nm can be distinguished in the steady-state PL spectrum of aqueous solutions for 2416SL at concentrations of 10^−4^ M (Figure 3b, curve 1). The position of the maximum of the first PL band corresponds to the S_1_(0) → S_0_(0) transition of the molecular solution, and the second maximum reflects the S_1_(0) → S_0_(1) transition of the H-aggregate, as this band is getting dominant in the high concentration solutions. An increase in the concentration from 5·10^−6^ M to 10^−4^ M leads to a significant drop in the intensity of the purely electronic S_1_(0) → S_0_(0) transition, and the intensity of the 594 nm band of the S_1_(0) → S_0_(1) transition relatively increases (Figure 3b, curve 1). At the concentration increased to 10^−3^ M, only a band with a maximum of 589 nm and a weak shoulder at 626 nm is observed in the PL spectrum (Figure 3b, curve 2).

The absorption spectrum of the aqueous solution of 2416SL (8·10^−2^ M) changes significantly with a temperature drop from 297 K (Figure 3a, curve 3) to 77 K (Figure 3a, curve 4). In the absorption spectrum at 77 K, the intensity of absorption at a longer wavelength increases dramatically and a new band of 571 nm appears. The presence of this band at low temperatures can be associated with structural changes in H-aggregates, a manifestation of their excitonic properties and the formation of low-temperature CTEs.

For the 10^−3^ M solution at 4.2 K, the intensity of the PL bands at 589 and 626 nm decrease to practically zero, and only bands with maxima at 685 and 745 nm are observed in the PL spectrum (Figure 3b, curve 3). The dramatic reduction of the 589 and 626 nm bands is characteristic of excimer emission [12]. In solutions, 2416SL molecules form elongated disc-like aggregates and, in such one-dimensional molecular aggregates, the formation of excimers has already been established [12]. Excimers should not be confused either with CTE states, which involve significant charge transfer between molecules, or with FEs, which characterize the coherent excitation of a crystal. An excimer is an optically excited dimer stabilized by resonance interaction. A necessary condition for the formation of excimers is a small distance between molecules, which is usually achieved due to effective π-stacking [16] and the convergence of molecules in an excited state. The ground state of excimers is antibonding; therefore, excimers have no direct absorption in the ground state and must be excited by energy transfer. The excimer radiation is characterized by a broad structureless band of PL. In 2416SL nanoaggregates, as in pyrene and α-perylene, several types of excimers can be realized [12]. In our case, the 2416SL excimers are featured by PL bands of 589 and 626 nm.

**Figure 4:**
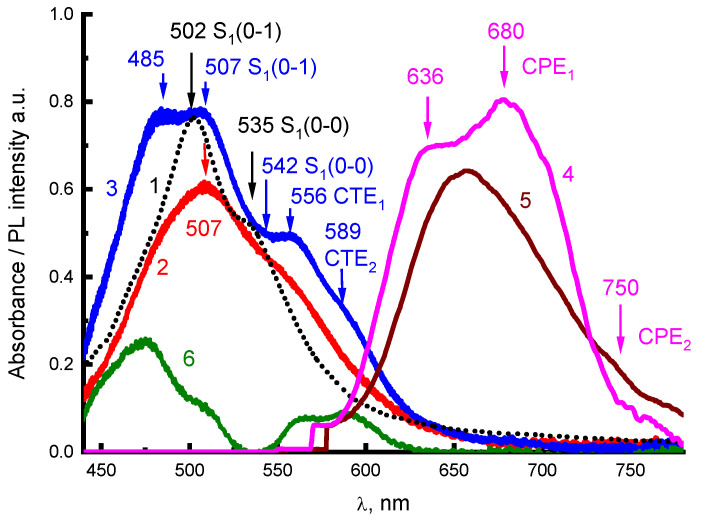
Absorption (1–3) and steady-state PL (4,5) of aqueous solution (1) of 2416SL at the concentration of 5·10^−6^ M (1) and 2416SL films before (2,5) and after thermal annealing at 470 K (3,4); differential spectrum, Δ, (6) obtained by subtraction of the spectrum (2) from the spectrum (3). *λ*_e_ = 405 nm; T = 296 K.

Changes in the PL spectra of the aqueous solutions at different concentrations can be associated with a manifestation of collective excitations of FEs, excimers, and CTEs in H-aggregates [7,12]. For molecular aggregates of perylene derivatives, CTEs play a significant role in PL [6,7,12,24,25,26,29]. Comparative analysis of absorption and PL spectra for 2416SL in solutions and condensed state allows us to determine the nature of these collective excitations. Figure 4 shows the spectra of absorption (Figure 4, curves 2,3) and steady-state PL (Figure 4, curves 4,5) of 2416SL films, before (Figure 4, curves 2,5) and after thermal annealing (Figure 4, curves 3,4). The absorption spectra of the films after thermal annealing (Figure 4) are more structured than the absorption spectra of nanoaggregates in solutions and are more like the absorption spectra of PTCDA films [6,7]. Such spectral changes can be associated with structural changes in the aggregates after thermal annealing. In the spectra of thermally annealed films, clearly expressed CTE absorption maxima at 556 (CTE_1_) and 589 nm (CTE_2_) appear (well evidenced by differential spectrum in Figure 4, curve 6). These maxima correspond to Franck-Condon’s non-relaxed optical CTE states [14,15]. The PL bands, which correspond to the emission of relaxed exciton states, have maxima at 636, 680, and 750 nm. The nature of the absorption and PL spectra in Figure 4 will be analyzed below.

CTE occupying an intermediate place in the classification of excitons based on their internal structure have the charge or its part transferred to a neighboring molecule due to photoinduced electron transfer [13,14,15]. With incomplete charge transfer, the wave function of the resulting state can be delocalized within two or more molecules and have both excitonic and ionic features. If the excitonic character prevails, CTE can coherently move along the crystal. Unrelaxed CTEs are formed directly upon optical excitation and appear in the absorption spectra. Due to a large static dipole moment (up to 25 Debye on the nearest molecules), CTEs can be a cause of a large nonlinear second-order polarizability and a strong electroabsorption. CTEs have a strong tendency to self-localize. They polarize the surrounding molecules, which leads to the relaxation of the crystal lattice into a new equilibrium. If the time of CTE excitation at the lattice nodes is longer than the lattice relaxation time, the excitation is accompanied by local deformation of the lattice and the formation of the excitonic polaron—CPE [14]. Relaxed molecular-polaron excitons (CPE) are a characteristic feature of molecular crystals. CPEs appear as intermediate states in the processes of photogeneration and radiative recombination of CTE states.

In addition to excimer radiation in the long-wavelength region of the PL spectrum for concentrated solutions of 2416SL (Figure 3), weakly intense bands of 680 and 750 nm can be distinguished. These bands are also observed in the spectra of 2416SL films at room temperature (Figure 4). When the 2416SL solution is cooled to 4.2 K, the PL intensity in this spectral region increases significantly and the bands at 685 and 745 nm appear in the spectra (Figure 3b, curve 3). The PL emission of the bands at 680 and 750 nm can be associated with CPE_1_ and CPE_2_, respectively.

Electronic states in quasi-one-dimensional molecular crystals of the PTCDA type with a strong overlap of molecular orbitals were comprehensively analyzed [3,6,7,12,24,25,26,29]. In such quasi-one-dimensional crystals, due to the small, less than 0.35 nm, intermolecular distance, there is a strong overlap of the π-orbitals of neighboring molecules. In such crystals, the difference between FE and CTE energies becomes small, and their strong mixing determines the nature of the lowest exciton states [7,13,30,31]. As soon as the energy difference between CTE and FE becomes small, both types of excitons can interact, and new mixed excitonic states are formed. At FE and CTE being close in energy, the FE band admixes some CTE states and shifts down, and CTE also shifts up, acquiring some energy of the FE state, and becomes optically allowed. These mixed FE-CTE states exhibit the properties of two types of excitons: FE provides a high oscillator strength, and CTEs lead to high sensitivity in external electric fields. Such exciton mixing can also result in a noticeable transition dipole for CTE [7]. This can explain the spectral dependence of the PTCDA films on their thickness in the range of 0.3–10 nm when the effects of quantum confinement become important [32]. In finite chains, simultaneously with excitonic “bulk” states, “surface” states can also arise [33]. The “surface” states are localized at the end of the chain and can be shifted to the blue or red region of the spectrum compared to the bulk states. Thus, PL in 2416SL nanoaggregates can be caused by direct excitation due to the borrowing of some transition oscillator strength from intense transitions with subsequent radiative recombination or thermally activated decay into free charge carriers. In 2416SL having a strong tendency for mixing of FEs and CTEs, the CTEs play a significant role in PL emission.

Registration of the dependence of the PL intensity on the decay time *t* was carried out for radiation wavelengths *λ_EM_* = 550 and 590 nm corresponding to molecular and H-aggregate emissions, respectively. The PL decays for *λ_EM_* = 550 nm at the concentration of 10^−4^ M are well described by a single-exponential function and feature molecular emission. The kinetics of PL decay for *λ_EM_* = 590 nm at the concentration of 10^−3^ M have a good fit by a biexponential function with fast and slow components of lifetimes *τ*. This can be attributed to the emission of molecular and aggregated forms of 2416SL. Table 3 shows the values of *τ* calculated for the PL kinetics of 2416SL.

**Figure 5:**
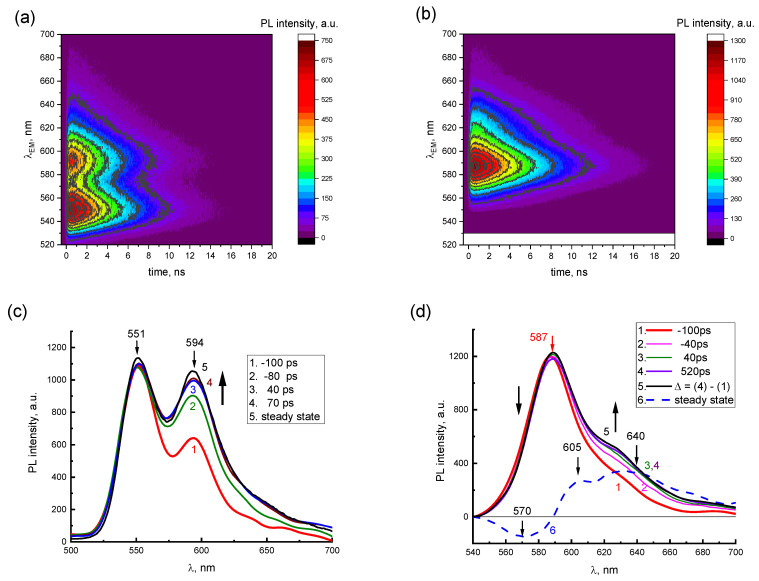
TRES maps (**a**,**b**) and instantaneous PL spectra at various delay times (**c**,**d**) of aqueous solutions of 2416SL for two concentrations 10^−4^ M (**a**,**c**) and 10^−3^ M (**b**,**d**). The following delay times are presented: (**c**) −100 (1), −80 (2), 40 (3), 70 ps (4); (**d**) −100 (1), −40 (2), 40 (3), 520 ps (4). The steady-state spectra (5) are shown as a reference. (**d**) Differential spectrum, Δ, (6) is obtained by subtraction of spectrum (1) from spectrum (4). *λ*_e_ = 405 nm; T = 296 K.

The presence of two emission components in the PL spectra of concentrated 2416SL solutions is demonstrated in TRES maps (Figure 5a,b) and instantaneous PL spectra (Figure 5c,d). In TRES maps, the dependence of the PL intensities vs. *t* and *λ_EM_* for aqueous solutions of 2416SL at the concentrations of 10^−4^ (Figure 5a) and 10^−3^ M (Figure 5b) is shown. PL emission wavelengths (520–700 nm) and delay times (0–20 ns) are plotted on the vertical and horizontal axes, respectively, whereas PL intensity is a function of color in relative units. For 2416SL concentration of 10^−4^ M (Figure 5a), two PL bands with maxima at 551 and 594 nm can be distinguished in the TRES map. When the concentration of 2416SL increases to 10^−3^ M (Figure 5b), the intensity of the band at 551 nm decreases significantly, and only the band at 589 nm remains.

In Figure 5c,d, instantaneous PL spectra are presented for aqueous solutions of 2416SL at various delay times *t* and concentrations, using the data of TRES maps in Figure 5a,b. The instantaneous PL spectra are normalized by the most intense PL bands in the steady-state spectra. These are bands with emission maxima at 551 nm and 589 nm for the concentration of 10^−4^ (c) and 10^−3^ M (d), respectively. Negative values of delay times mean that instantaneous PL spectra were recorded at the leading edge of the laser pulse. It can be seen from Figure 5c that at short delay times of −100 ps, the instantaneous PL spectra are similar to the molecular spectra of Figure 3, and already at delay times of 40 ps, they coincide with the steady-state PL spectra (Figure 5c, curves 3,5), which is also manifesting contribution of the aggregated form of 2416SL.

Figure 5d shows the instantaneous PL spectra for aqueous solutions of 2416SL at the concentration of 10^−3^ M, which reflect the time dependence of the instantaneous spectra of the aggregated form of 2416SL. In the time delay of around 40 ps, the instantaneous PL spectra reach an equilibrium value and become similar to the steady-state PL spectra (Figure 5d, curves 3,5). A relative decrease in PL on the long-wavelength side of the 589 nm band is also observed. The presented difference spectrum (Figure 5d, curve 6), obtained by subtracting the instantaneous spectra at the time delay of 520 and −100 ps (Figure 5d, curves 4,1), has bands with maxima at 570, 605, and 640 nm. Analysis of the instantaneous PL spectra (Figure 5) and the lifetimes (Table 3) allows ones to conclude that the band maxima at 551, 589, 626, 685 and 745 nm are of different natures. PL in these bands has different lifetimes and differs in temperature dependence.

### 3.2. Dispersions of CdTe QDs

**Figure 6:**
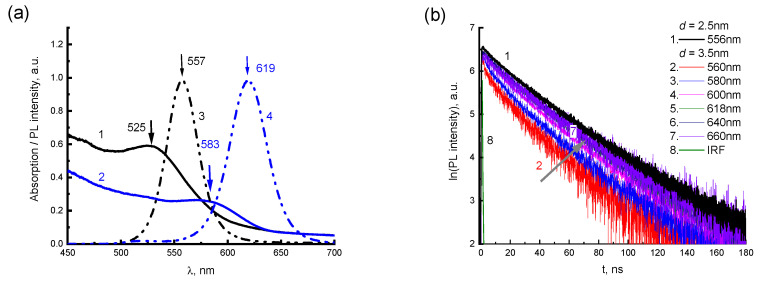
(**a**) Absorption (1,2) and PL (3,4) spectra of aqueous dispersions of CdTe QDs with a diameter of *d* = 2.5 (1,3) and 3.5 (2,4) nm. (**b**) PL kinetics at various *λ_EM_* for aqueous dispersions of CdTe QDs with a diameter of 2.5 (1) and 3.5 (2–7) nm. *λ*_e_ = 405 nm. T = 296 K.

In Figure 6a, absorption and PL spectra are shown for initial aqueous dispersions of CdTe QDs with a diameter of *d* = 2.5 nm (curves 1,3) and 3.5 nm (curves 2,4). The spectra evidence the positions of exciton transitions for CdTe QDs. In the absorption spectra of CdTe QDs, bands with maxima at 525 and 583 nm can be distinguished, which correspond to the excitonic absorption of the QDs with *d* = 2.5 and 3.5 nm, respectively [34]. Exciton emission bands with maxima at 557 and 619 nm can be distinguished in the PL spectra (Figure 6, curves 3,4) of these QDs. As the size of QDs increases, the spectra of exciton absorption and emission shift to the long-wavelength side (Figure 6a), having a clear manifestation of the quantum-size effect [34].

Measurements of time-resolved PL spectra for the dispersions of CdTe showed that, for QDs with a diameter of 2.5 nm, the band at 557 nm does not change with delay time. This allows us to confirm that the studied dispersions of CdTe QDs at *d* = 2.5 nm have a very narrow distribution of their sizes. For CdTe QDs with a diameter of 3.5 nm, the time-resolved PL (TRES) depends on the time delay. At small delay times, in addition to the dominant emission band of 619 nm, an additional shoulder was observed on the short-wavelength side of the PL spectra evidencing broader distribution of sizes for CdTe QDs in the range of 3.5 nm diameter.

The dependence of PL lifetimes for CdTe QDs on their diameter (*d*) and *λ_EM_* is shown in Figure 6b and Table 4. Kinetics of PL decays for the dispersions of CdTe QDs at different *λ_EM_* are well described by a three-exponential function: I_PL_(t)= A_1_e^−t/τ^_1_ +A_2_ e^−t/τ^_2_ +A_3_ e^−t/τ^_3_. As can be seen from Table 4, the shorter *λ_EM_* is, the faster the PL lifetime is for CdTe QDs at *d* = 3.5 nm. The PL spectra for the 3.5 nm CdTe QDs are dominated by components with lifetimes from 31 to 62 ns.

### 3.3. Mixing of Excitons in Hybrid Systems of CdTe-2416SL

QDs of CdTe stabilized by thioglycolic acid are negatively charged and can adsorb on the surface positively charged molecules by Coulombic attraction. 2416SL molecules have a positive charge (Figure 1) and can be attached to the surface of CdTe due to electrostatic interaction.

**Figure 7:**
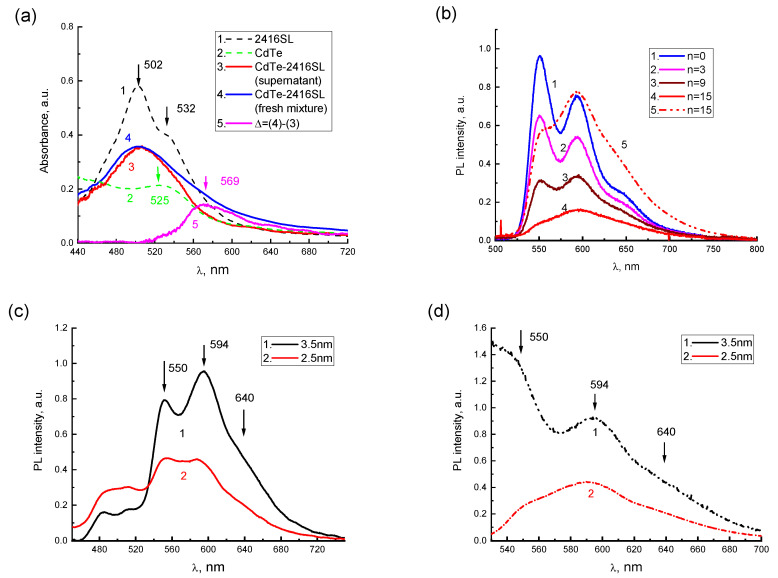
(**a**) Absorption spectra of the aqueous solution of 2416SL (5·10^−5^ M) (1), initial dispersion of CdTe QDs (*d* = 2.5 nm) (2), the mixture of CdTe-2416SL at *n* = 15 (3,4) in the form of supernatant (nanoaggregates precipitated) (3) and freshly mixed having nanoaggregates in the dispersion (4). Differential spectrum, Δ, (5) obtained by subtraction of the spectrum (3) from the spectrum (4). (**b**) PL quenching dynamics for the aqueous solution of 2416SL (5·10^−5^ M) with fresh admixing of CdTe QDs (*d* = 2.5 nm) dispersions at *n* = 0 (1), *n* = 3 (2), *n* = 9 (3), *n* = 15 (4,5); *λ*_e_ = 385 (1–4) and 510 (5) nm. (**c**,**d**) PL spectra for (supernatant) mixtures of CdTe-2416SL at *n* = 8 for CdTe QDs with *d* = 3.5 nm (1) and 2.5 (2) nm; *λ*_e_ = 385 (**c**) and 510 nm (**d**). T = 296 K.

After the mixture of CdTe-2416SL at *n* = 15, a broad absorption band (Figure 7a, curve 3) with a maximum at 504 nm was observed. In Figure 7a, the absorption spectra of the aqueous solutions of neat 2416SL (Figure 7a, curve 1) and initial dispersions of CdTe (Figure 7a, curve 2) are shown as a reference. A part of the formed CdTe-2416SL nanoaggregates gradually precipitate. To analyze the spectral features of the nanoaggregates, absorption spectra of CdTe-2416SL supernatant without precipitate (Figure 7a, curve 3) and freshly mixed CdTe-2416SL with all the nano-aggregations present (Figure 7a, curve 4) were compared. The spectrum of freshly mixed CdTe-2416SL represent a superposition of the absorption of the supernatant and precipitated aggregate. The differential spectrum, Δ, (Figure 7a, curve 5) obtained by subtraction of the supernatant spectrum from freshly mixed CdTe-2416SL spectrum demonstrate spectral features of the CdTe-2416SL nanoaggregates. The differential absorption spectrum, which is characterized by a new absorption band with a maximum at 569 nm, can be associated with light absorption by CdTe-2416SL nanoaggregates. The new absorption band that appears in the hybrid structures of CdTe-2416SL (Figure 7a) can be associated with the hybridization of CTE and WME at the interface of 2416SL and CdTe.

The addition of the CdTe dispersions to the solution of 2416SL resulted in significant quenching of 2416SL emission with the most intense relative quenching of PL in the 550 nm band (Figure 7b). The band with a maximum at 595 nm featuring H-aggregated 2416SL remains in the PL spectrum of CdTe-2416SL mixtures and becomes more prominent upon excitation with *λ*_e_ = 510 nm (Figure 7b, curve 5) evidencing efficient absorption responsible for this emission. This behavior of the absorption and PL spectra can be associated with the formation of CdTe-2416SL nanoaggregate structures, as the observed emission is different to the features of H-aggregate and molecular PL of neat 2416SL. Importantly, the PL of CdTe QDs, which have a high quantum yield in the dispersions, was not observed. Strong quenching of QDs emission can be associated with the hybridization of WME in the CdTe QDs with the CTE states at the interface of 2416SL and CdTe and the formation of mixed exciton states—CTE-WME.

PL spectra of CdTe-2416SL nanoaggregates based on CdTe QDs with a diameter of 3.5 and 2.5 nm (Figure 7c,d) are similar featuring intense PL bands with maxima at 550, 594 and 640 nm. The bands are associated with the formation of CdTe-2416SL nanoaggregates. The difference between these spectra can be attributed to the change of spectral positions of excitonic transitions for QDs of various diameters.

**Figure 8:**
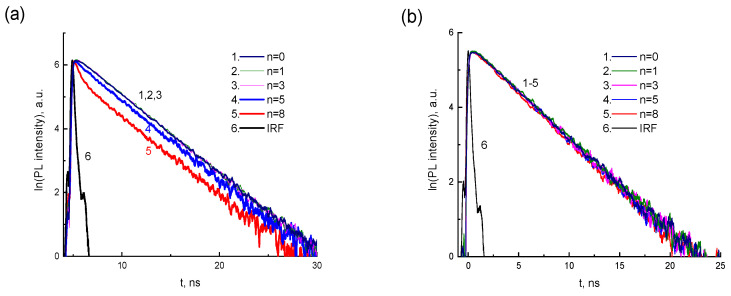
PL kinetics for CdTe-2416SL mixtures at *λ_EM_* = 550 nm (**a**) and 620 nm in (**b**) depending on the concentration of CdTe (*d* = 3.5 nm) QDs (*V_n_*): *n* = 0 (1), *n* = 1 (2), *n* = 3 (3), *n* = 5 (4), *n* = 8 (5); *λ*_e_ = 405 nm. T = 296 K.

Figure 8 shows the PL kinetics of CdTe-2416SL mixtures for two *λ_EM_* (550 nm in Figure 8a and 620 nm in Figure 8b) depending on the concentration of CdTe QDs. The measured PL lifetimes for the above wavelengths are shown in Table 5. According to the PL kinetics, the PL lifetime in the 620 nm band does not depend on the CdTe concentration. The PL intensity and lifetime in the 550 nm band decrease with increasing CdTe concentration, which can be associated with the processes of CdTe-2416SL formation and energy transfer in them. Förster resonant energy transfer (FRET) mechanism could be involved in studied CdTe-2416SL nanostructures.

2416SL-CdTe nanocomposites arise due to the electrostatic interaction between the columnar nanoaggregates of 2416SL and CdTe QDs. A new band with a maximum of 569 nm appears in the absorption spectra of the mixtures (Figure 7a, curve 5), which can be associated with the mixed exciton states—CTE-WME. The band with a maximum at 594 nm in the PL spectra of mixtures can also be attributed to the emission of mixed CTE-WME states. Such states arise due to the close position of CTE of 2416SL and WME in CdTe QDs. Electronic states and resonant energy transfer in hybrid nanostructures containing organic and inorganic semiconductor materials were studied before [35,36,37,38,39]. The high efficiency of non-radiative energy transfer from semiconductor nanostructures (quantum wells/QDs) to organic material with overlapping electronic excitation spectra has been demonstrated [36,37,38,39]. The time of energy transfer for WME to organic matter is less than the exciton lifetime in the absence of an organic coating [35,36,37,38,39]. In our case, significant changes of the PL spectra in the emission ranges of 2416SL and CdTe QDs are observed for the mixtures. The intensity and lifetime of PL in the emission band of CdTe-2416SL nanoaggregates (550 nm band) decrease with increasing CdTe concentration, which can be associated with FRET and formation of CdTe-2416SL nanostructures.

For FRET, the rate of energy transfer depends on the degree of overlapping of the PL spectrum of the donor and the absorption spectrum of the acceptor, the mutual orientation of the transition dipole moments, and on the distance *R* between the interacting molecules [40,41]. As a result of FRET, the fluorescence quantum yield of the donor *φ_d_* and the lifetime of the excited state of the donor *τ_d_* decrease compared to the intrinsic radiation time *τ_D_*, since an additional channel for reducing the population of the excited state of the donor with the migration constant *k_m_* appears. If the donor and acceptor molecules are at a distance *R* ≠ *R_0_* from each other, then the ratio between the characteristic migration time *τ_m_* = *k_m_*^−1^ and the intrinsic radiation lifetime of the excited state of the donor:

*τ_m_* = *τ_D_* · (*R*/*R*_0_)^6^

where *R_0_* (Förster radius) is the characteristic distance, at which the probability of FRET is equal to the probability of spontaneous fluorescence of the donor molecule and is determined by the condition *k_m_·τ_D_* = 1. In the first case of potential FRET, the 2416SL with absorption of 502 and 532 nm and PL emission peaking at 549 and 591 might be a donor, and the QDs would act as an acceptor. Absorption levels of QDs with *d* = 2.5 nm are not having much of overlap for the above FRET conditions to be met properly. However, for 2416SL adsorbed on the QDs with a diameter of 3.5 nm, the above conditions for energy transfer are reasonably satisfied. The emission band of 2416SL overlaps with the absorption band of 3.5 nm CdTe QDs having a maximum of about 583 nm. Furthermore, it needs to be admitted that there is practically no PL emission from the QD levels for the mixtures due to formation of CTE-WME states with low quantum yield. Another case of potential FRET process might involve CTE states of 2416SL (absorption maxima at 556 and 589 nm) that could act as acceptors of energy from QDs levels. The photon energy absorbed by CTEs could quickly relax to CPE levels of emission, but due to CTE-WME hybridization the absorbed energy more likely to relax on the CTE-WME levels as these levels have much longer lifetime, and the emission would be observed from these mixed levels. Therefore, the FRET from the QDs to CTE is unlikely process. Overall, the hybrid CdTe-2416SL nanostructures with strong quenched emission of QDs might be applied in dissociative sensing. Such sensors would work by enabled interaction of the perylene dye with an analyte, leading to the dissociation of the nanostructures and an emergence of strong PL emission of the QDs. Current research provides a fundamental understanding for the emission of the hybrid CdTe-2416SL nanostructures and further studies toward sensing would be pursued to provide clear insight into the above applications.

**Figure 9:**
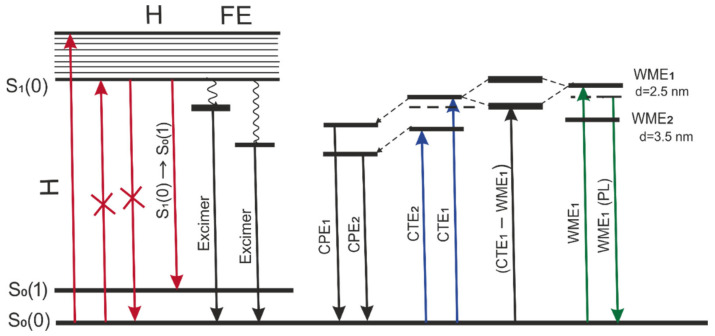
Energy diagram of exciton transitions for 2416SL in the condensed phase and CdTe-2416SL nanoaggregates. More details about each transition are summarized in Table 6.

The energy diagram is proposed in Figure 9 to elucidate various types of exciton transitions for 2416SL in the condensed phase and mixed excitons in 2416SL nanocomposites with CdTe QDs. The nature of excitonic transitions has been discussed above, and the diagram is aiming to group the transitions and visualize the complexity of studied transitions. The first group is associated with FE transitions in 2416SL H-aggregates that occur in the solutions and films. In addition to FE, at optical Frank-Condon transitions, it is possible to excite an unrelaxed electron-polaron pair: excitons with charge transfer (CTE_1_ and CTE_2_). Relaxed molecular-polaron pairs (CPE_1_ and CPE_2_) appear as intermediate states in the processes of photogeneration and radiative recombination of the CTEs. In the hybrid 2416SL-CdTe nanostructures, the unrelaxed CTE_1_ state of the aggregated 2416SL and the WME of CdTe QDs form mixed exciton states. As can be seen from the diagram, the interaction of exciton states leads to the appearance of new levels above the bottom of the exciton zone for CdTe WME and the exciton zone of CTE_1_. The mixed exciton transition is indicated on the diagram as CTE_1_-WME_1_ transition. The emission of hybrid 2416SL-CdTe nanostructures (e.g., 594 nm) strongly overlaps with the emission of 2416SL aggregates and is not included in the diagram. Table 6 summarizes the nature and values of exciton transitions for absorption and PL in the studied systems.

## 4. Conclusions

Comprehensive studies of the optical properties of water-soluble perylene derivative, 2416SL, were carried out using absorption spectroscopy and techniques of steady-state and picosecond time-resolved PL. Comparison of the absorption and PL spectra of 2416SL in solutions and films revealed the nature of the molecular and aggregated excitonic states. Spectral and lifetime analysis allowed us to identify optical bands of FE, CTE and mixed FE-CTE states. Furthermore, the pathways of non-radiative and radiative relaxation of the indicated collective electronic excitations were determined in steady-state and picosecond time-resolved PL spectra. The emission of excimer and localized polaron states with charge transfer has been identified.

In the aqueous mixtures of 2416SL and CdTe QDs, the aggregation of 2416SL molecules on the surface of CdTe results in the hybridization of CTE and WME and the formation of mixed CTE-WME states. The new absorption and PL bands that appear in the mixtures of CdTe-2416SL as well as strong quenching of QDs emission are associated with such hybridization. FRET from the dye to the CdTe QDs in CdTe-2416SL nanostructures has been analyzed proving its feasibility for hybrid nanostructures made of CdTe QDs of 3.5 nm in diameter. The energy diagram of possible exciton transitions leading to mixed excitons in CdTe-2416SL nanocomposites is proposed to help in understanding the nature of excitonic transitions. Learning more about the fundamental nature of mixed excitons at the interface of organic and inorganic nanostructures makes us a step closer to the application of excitonic elements in molecular electronics and optoelectronics.

## Figures and Tables

**Table 1:** Positions of electronic absorption band maxima for solutions of 2416SL.

Solution	Transitions (nm/cm^−1^)		
	S_0_(0) → S_1_(0)	S_0_(0) → S_1_(1)	S_0_(0) → S_1_(2)
DMSO	530/18,870	495/20,200	464/21,550
water	532/18,800 *	502/19,920	471/21,220

* The transition is estimated from the comparison of the absorption spectra of aqueous and DMSO solutions, the values of their vibrational repetitions, and Stokes shifts.

**Table 2:** Positions of PL band maxima of 2416SL solutions.

Solution	Transitions (nm/cm^−1^)		
	S_0_(0) ← S_1_(0)	S_0_(0) ← S_1_(1)	S_0_(0) ← S_1_(2)
DMSO	545/18,340	586/17,070	633/15,800
water	549/18,190	591/16,920	639/15,650

**Table 3:** PL lifetimes of aqueous solutions of 2416SL for various concentrations and *λ_EM_*; *λ*_e_ = 405 nm; T = 296 K.

2416SL Concentrations	*λ_EM_*, nm	*τ*_1_, ps	%	*τ*_2,_ ps	%	χ^2^
10^−4^ M	550	-		4050		1.162
10^−3^ M	590	1710	3	4760	97	1.068

**Table 4:** PL lifetimes for aqueous dispersions of CdTe QDs depending on their diameter *d* and *λ_EM_*. *λ*_e_ = 405 nm. T = 296 K.

CdTe Diameter	*λ_EM_*, nm	*τ*_1,_ ns	%	*τ*_2,_ ns	%	*τ*_3,_ ns	%	χ^2^
2.5 nm	556	2.3	1.3	20	38.7	42	60	1.355
3.5 nm	560	0.160	2.3	7.7	11	31	66.7	0.936
	580	0.150	1.7	13.7	24.3	40.7	74	1.077
	600	0.180	1.1	17	29	46	69.9	1.230
	618	0.160	1	20	32	48	67	1.065
	640	0.137	0.8	22.6	34.2	49	65	1.072
	660	0.134	0.6	14	9.4	62	90	1.058

**Table 5:** PL lifetimes for CdTe-2416SL mixtures depending on *λ_EM_* and the concentration of CdTe (*d* = 3.5 nm) QDs (*n*); *λ*_e_ = 405 nm. T = 296 K.

*λ_EM_*, nm	*n*	*τ*_1_, ps	%	*τ*_2_, ps	%	χ^2^
550	1, 3			4040		0.967
550	8	350	11	3900	89	1.037
620	1, 3			4450		1.105
620	8			3770		1.037

**Table 6:** Exciton transitions for 2416SL in the condensed phase and CdTe-2416SL nanoaggregates.

#	Nature of the Transitions		*λ*, nm	E, eV	Comments
1	S_0_ (0) → S_1_(1), FE	Absorption	507	2.44	
2	S_0_ (0) → S_1_(0), FE	Absorption	542	2.29	forbidden
3	S_1_(0) → S_0_(0), FE	PL	550	2.26	forbidden
4	S_1_(0) → S_0_(1), FE	PL	594	2.09	
5	Excimer-1, FE	PL	589	2.11	
6	Excimer-2, FE	PL	636	1.95	
7	CTE_1_	Absorption	556	2.23	
8	CTE_2_	Absorption	589	2.11	
9	CPE_1_	PL	680	1.82	
10	CPE_2_	PL	750	1.65	
11	WME_1_	Absorption	525	2.36	*d* = 2.5 nm
12	WME_2_	Absorption	583	2.13	*d* = 3.5 nm
13	WME_1_	PL	557	2.23	*d* = 2.5 nm
14	WME_2_	PL	619	2.00	*d* = 3.5 nm
15	CTE_1_–WME_1_	Absorption	569	2.18	

## Data Availability

The data presented in this study are available on request from the corresponding author.
